# Effect of Recycled Polypropylene Fibers on the Compressive and Tensile Properties of Concrete: Fiber Type, Aspect Ratio and Volume Fraction

**DOI:** 10.3390/polym18151825

**Published:** 2026-07-25

**Authors:** Zhongli Xu, Chunsheng Hu, Yang Zhao, Hongxiang Wu, Yingtao Zhan, Wei Wang, Na Li, Ping Jiang

**Affiliations:** 1 State Key Laboratory of Intelligent Deep Metal Mining and Equipment, School of Civil Engineering, Shaoxing University, Shaoxing 312000, China; 2 Zhejiang Key Laboratory of Rock Mechanics and Geohazards, School of Civil Engineering, Shaoxing University, Shaoxing 312000, China; 3 Tongchuang Engineering Design Co., Ltd., Shaoxing 312000, China; 4 Zhejiang Jinggong Steel-Building Group Co., Ltd., Shaoxing 312000, China; 5 CTA High-Tech Fiber Co., Ltd., Shaoxing 312000, China

**Keywords:** fiber concrete, recycled polypropylene fiber, compressive strength, splitting tensile strength

## Abstract

This study aimed to evaluate whether short-cut recycled polypropylene (RPP) fibers can serve as an effective and sustainable alternative to virgin polypropylene (PP) fibers for improving the mechanical performance and crack-control behavior of concrete. For this purpose, PP and RPP fibers with two lengths, 3 mm and 6 mm, and three volume fractions, 0.1%, 0.2%, and 0.3%, were incorporated into C35 concrete. Compressive strength, splitting tensile strength, apparent stress–strain response, slump, failure morphology, and microstructural characteristics were examined at curing ages of 7 and 28 days. The results showed that both PP and RPP fibers improved crack distribution and failure integrity, while the mechanical enhancement strongly depended on fiber type, length, and dosage. Among all mixtures, RPPC3-0.1 exhibited the best overall performance; at 28 days, its compressive strength and splitting tensile strength were approximately 6% and 24% higher than those of plain concrete, respectively. These findings suggest that low-dosage short RPP fibers can effectively enhance the tensile-related performance of concrete while providing a feasible pathway for the value-added reuse of polypropylene waste.

## 1. Introduction

Concrete is one of the most widely used construction materials globally, forming the backbone of critical infrastructure such as tunnels, bridges, and highways [1,2]. Annual global consumption of concrete exceeds 30 billion tons and continues to rise steadily [3]. However, ordinary concrete is inherently brittle and has limited tensile resistance. Once microcracks initiate, particularly under tensile or flexural loading, they can propagate rapidly and develop into macrocracks, thereby reducing structural integrity and limiting durability and service performance [4]. To mitigate these limitations, fiber reinforcement has been widely adopted as an effective strategy to improve crack resistance, tensile-related performance, and toughness of cementitious composites [5].

In fiber-reinforced concrete, commonly used fibers include steel fibers, polypropylene fibers, glass fibers, and natural fibers [6]. Natural fibers have also been studied as reinforcing materials for concrete and mortar mixtures due to their renewability, low density, low cost, and relatively low environmental impact. These fibers can improve crack resistance and toughness to a certain extent and have a certain toughening effect on sustainable cement-based composites [7,8]. Polypropylene (PP) fibers have attracted much attention due to their low density, corrosion resistance, good chemical stability, and good compatibility with concrete construction [9]. Previous studies have shown that PP fibers can inhibit the initiation and propagation of microcracks through fiber bridging, redistribute local stress, and reduce stress concentration, thereby delaying crack propagation and improving the ductility of concrete [10,11,12]. Therefore, the reinforcing effect of PP fibers is usually reflected not only in the improvement of strength, but also in the change in deformation response and failure mode.

In this context, compressive strength and splitting tensile strength are two fundamental indicators for evaluating the mechanical performance of fiber-reinforced concrete. Compressive strength reflects the load-bearing capacity of concrete under compression, whereas splitting tensile strength is closely associated with crack initiation and propagation under tensile stress [13]. In addition to these strength indices, the apparent stress–strain response provides useful supplementary information on deformation capacity and damage evolution during loading. Under compression, fibers may delay microcrack coalescence, alter the apparent strain response near peak stress, and help maintain specimen integrity after peak load. Under splitting tensile loading, the deformation response is closely related to crack initiation, crack propagation, and fiber bridging or pull-out across the splitting plane [14]. Therefore, combining strength results, apparent stress–strain behavior, and failure morphology can provide a more comprehensive understanding of the crack-control mechanisms of fiber-reinforced concrete.

Nevertheless, traditional PP fibers face growing constraints because they are derived from nonrenewable petroleum resources and are associated with relatively high production costs and limited environmental sustainability. These limitations restrict their large-scale application in sustainable construction. Against the background of carbon reduction and the development of green building materials, recycled polypropylene (RPP) fibers have gradually attracted attention owing to their potential advantages in resource utilization, cost reduction, and environmental impact [15]. Win et al. [16] incorporated recycled shredded disposable masks into mortar and observed modest improvements in compressive and tensile strength. Shariati et al. [17] used recycled carpet fibers to modify concrete and reported that the mechanical properties improved most significantly at a fiber volume fraction of 0.5%, whereas they decreased when the volume fraction increased to 0.75% and 1.0%. However, these waste-derived fiber materials still have limitations in terms of material composition and performance stability. For example, disposable masks may vary considerably in composition because of differences in brands and manufacturing processes, which can increase the dispersion of test results. Carpet fibers are often multi-polymer blended systems and may contain fillers, dyes, and other additives, making it difficult to achieve stable control of mechanical properties. In contrast, RPP fibers obtained through classified recycling and standardized processing of polypropylene waste can offer relatively uniform material characteristics and more stable performance.

Current research on RPP fiber-modified concrete has mainly focused on applications using relatively long fibers, typically 10–60 mm [18,19,20], whereas systematic investigations into short-cut RPP fibers, especially fibers with lengths of 3 mm and 6 mm, remain limited. In particular, the combined effects of fiber type, fiber length, and low fiber volume fractions on compressive strength, splitting tensile strength, apparent stress–strain response, failure morphology, and microstructural characteristics at different curing ages have not been sufficiently clarified. To address these gaps, this study incorporates PP and RPP fibers with two lengths, 3 mm and 6 mm, and three volume fractions, 0.1%, 0.2%, and 0.3%, into C35 concrete. Compressive strength, splitting tensile strength, apparent stress–strain response, workability, failure morphology, and microstructural characteristics were examined at 7 and 28 days. The results are expected to provide an experimental basis for the rational use of short-cut RPP fibers in concrete and to support the value-added reuse of polypropylene waste in sustainable construction materials.

## 2. Raw Materials and Sample Preparation

### 2.1. Raw Materials

**Figure 1:**
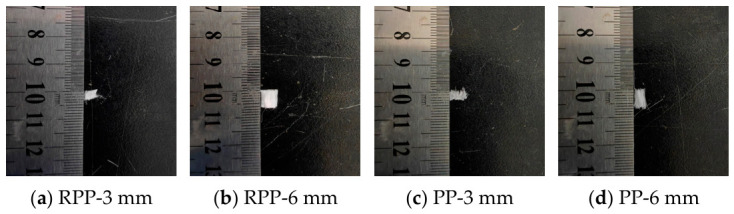
Comparison of length between PP and RPP fibers.

**Figure 2:**
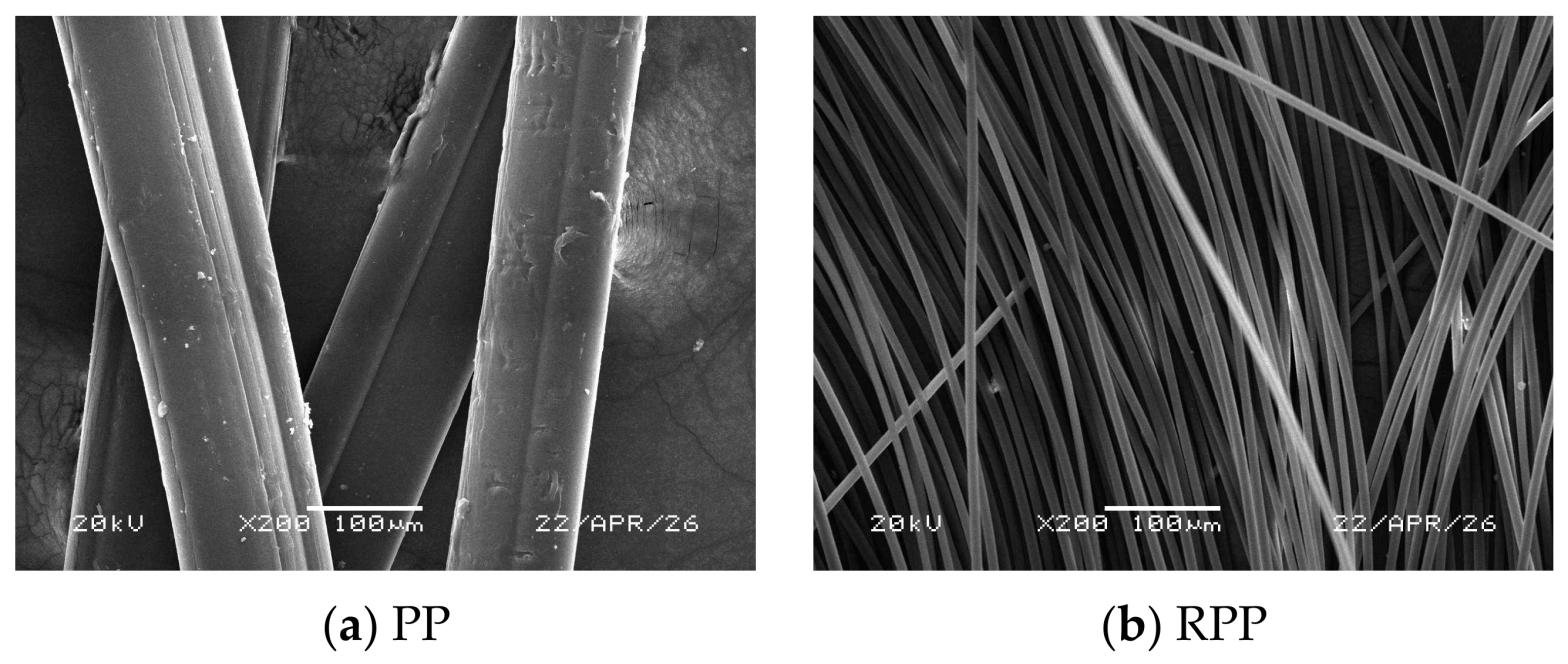
SEM images of different fibers.

The cement used in this study was P.O 42.5 ordinary Portland cement, supplied by Shaoxing Zhaoshan Building Materials Co., Ltd. (Shaoxing, China) The fine aggregate is natural river fine aggregate with a particle size of 0.5–2 mm, a fineness modulus of 2.66, a specific gravity of 2.6, and a water absorption rate of 1.1%, selected in accordance with the Standard for Technical Requirements and Test Method of Sand and Crushed Stone (or Gravel) for Ordinary Concrete (JGJ 52-2006) [21]. The coarse aggregate has a continuous gradation of 5–20 mm, a crushing index of 14.8%, a specific gravity of 2.7, and a water absorption rate of 0.9%; the particle size distributions of fine and coarse aggregates are shown in Table 1 and Table 2 below, compliant with the same standard. The mixing water was municipal tap water, meeting potability requirements for concrete production. The main source of RPPF used in this study is waste polypropylene textile materials. The extraction process is as follows: (1) Cut the cleaned raw materials into 20 × 20 cm samples, dry them in a 90 °C oven for 40 min, and weigh them. (2) Soak the dried samples in a pretreatment oil for 10 min, take them out, and then roll them evenly with a rolling mill to control the oil content to about 4%. After air drying, open them to obtain recycled polypropylene fibers. (3) Put the recycled polypropylene fibers into a twin-screw reprocessing machine and melt granulate them at 190–230 °C. (4) Make the masterbatch into continuous fibers with a diameter of 15 μm through melt spinning process, and cut them into 3 mm or 6 mm recycled polypropylene short fibers through a cutting machine. PPF was purchased through an online shopping platform. Its physical photo is shown in Figure 1, and its SEM image is shown in Figure 2. Its main physical properties are shown in Table 3.

### 2.2. Experimental Design and Sample Preparation

According to the Specification for Mix Proportion Design of Ordinary Concrete (JGJ 55-2011) [22], the target strength grade of concrete was set to C35. As detailed in Table 4, the reference mix proportion by mass was cement:coarse aggregate:fine aggregate:water = 1:3.1:1.78:0.5. Polypropylene (‌PP‌) and recycled polypropylene (‌RPP‌) fibers were incorporated at contents of ‌0.1%‌, ‌0.2%‌, and ‌0.3%‌ relative to the total concrete volume [23,24]. The specimens were cast into 100 mm × 100 mm × 100 mm cubes according to the standard curing procedure. There were 144 specimens in total, including 4 types of fibers, 3 dosages, 2 curing periods, 2 mechanical tests, and 3 parallel samples. In addition to the 12 reference groups, a total of 156 specimens were made in this experimental study. We used a standard naming system to identify each type of mixture. In the specimen label, “PPC” and “RPPC” indicate concrete containing virgin PP fibers and recycled PP fibers, respectively. The numerical suffix “3” or “6” indicates fiber length (in millimeters), while the decimal suffix “0.1”, “0.2”, or “0.3” indicates fiber volume fraction (in percentage). For example, PPC3-0.1 denotes concrete containing 3 mm virgin PP fibers at a volume fraction of 0.1%, whereas RPPC3-0.1 denotes concrete containing 3 mm recycled PP fibers at the same volume fraction. The control group of unreinforced plain concrete was labeled PC.

The sample preparation process was conducted in accordance with the Standard for Test Method of Performance on Ordinary Fresh Concrete (GB/T 50080-2016) [25]. To improve fiber dispersion and reduce agglomeration, the fibers were first dry-mixed with a small amount of fine aggregate. The friction and shearing action of the fine aggregate helped separate the lightweight fibers and weaken local entanglement. Subsequently, the coarse aggregate and the remaining fine aggregate were added and dry-mixed for 30 s, followed by cement addition and further dry-mixing for 60 s. Approximately two-thirds of the designed water was then added, and the mixture was wet-mixed for 120 s to achieve initial homogenization. The remaining water was added and mixed for another 60 s to ensure uniform dispersion of the components. After mixing, the fresh concrete was transferred to an iron tray and cast into oiled molds in three equal layers. Each layer was compacted 25 times using a tamping rod or vibrator to reduce entrapped air and ensure consistent compaction. The specimens were demolded after 24 h and then cured in a standard curing environment at 20 ± 2 °C and a relative humidity of not less than 95%. Mechanical tests were conducted after 7 and 28 days of curing.

### 2.3. Test Methods for Mechanical Properties

#### 2.3.1. Slump Test and Workability Evaluation

The slump test of fresh concrete was conducted in accordance with the Standard for Test Methods of Performance of Ordinary Fresh Concrete (GB/T 50080-2016) [25]. Before testing, the slump cone and base plate were moistened without standing water. The fresh concrete was placed into the slump cone in three layers, and each layer was uniformly tamped according to the standard procedure. After the top surface was leveled, the cone was lifted vertically within 3–7 s. For each mixture, three measurements were performed, and the mean value was reported.

#### 2.3.2. Compressive Strength Test

**Figure 3:**
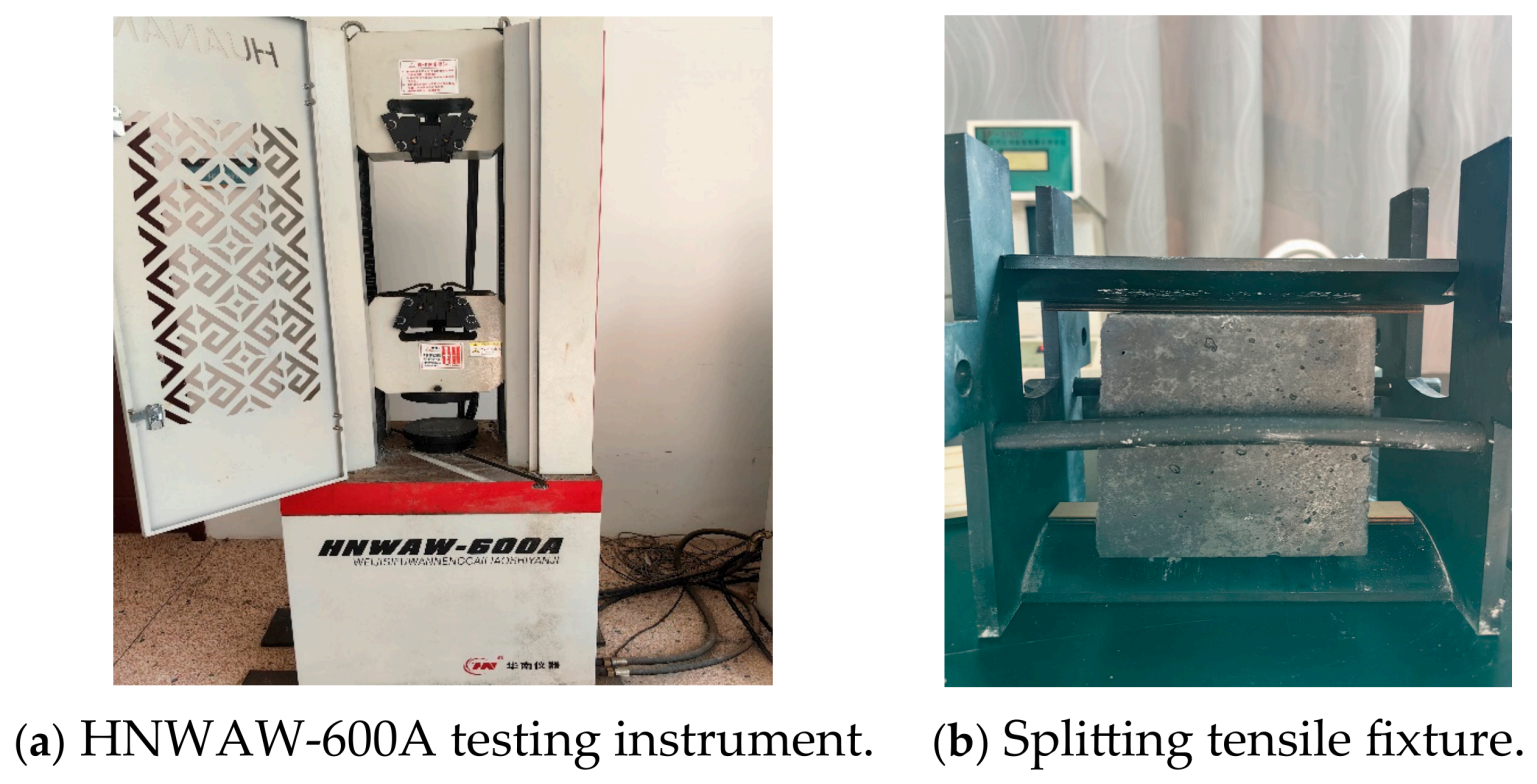
Testing instrument.

According to the “Standard for Test Methods of Physical and Mechanical Properties of Concrete” (GB/T 50081-2019) [26], compressive strength tests were conducted at 7 days and 28 days of curing. The test was conducted using an HNWAW-600A testing machine (Huaan Instrument Co., Ltd., Hangzhou, China), as shown in Figure 3a. During the compressive strength test, the specimen was continuously and uniformly loaded at a constant loading rate of 0.8 MPa/s until failure. Because 100 mm × 100 mm × 100 mm cubic specimens were used, a size conversion factor of 0.95 specified in GB/T 50081-2019 was applied to calculate the equivalent compressive strength. Three parallel specimens were used in each test group, and the arithmetic mean of the three measurement results was taken to reduce the test error. The obtained apparent stress–strain curves have certain limitations because no correction was applied for machine compliance, platen deformation, seating effects, or possible end-slip. Therefore, the strain values reported in this study should be regarded as apparent strain values rather than directly measured material strain.

#### 2.3.3. Splitting Tensile Strength Test

Splitting tensile strength tests were carried out at 7 and 28 days curing ages in accordance with the Standards for Test Methods of Concrete Physical and Mechanical Properties (GB/T 50081-2019) [26]. The same HNWAW-600A testing instrument (Figure 3a) was employed, with the addition of a standardized splitting tensile fixture (Figure 3b). A continuous and uniform load was applied at a constant rate until the specimen failure by splitting. The peak load at failure was recorded for subsequent calculation of splitting tensile strength.

## 3. Results

### 3.1. Performance Analysis

**Figure 4:**
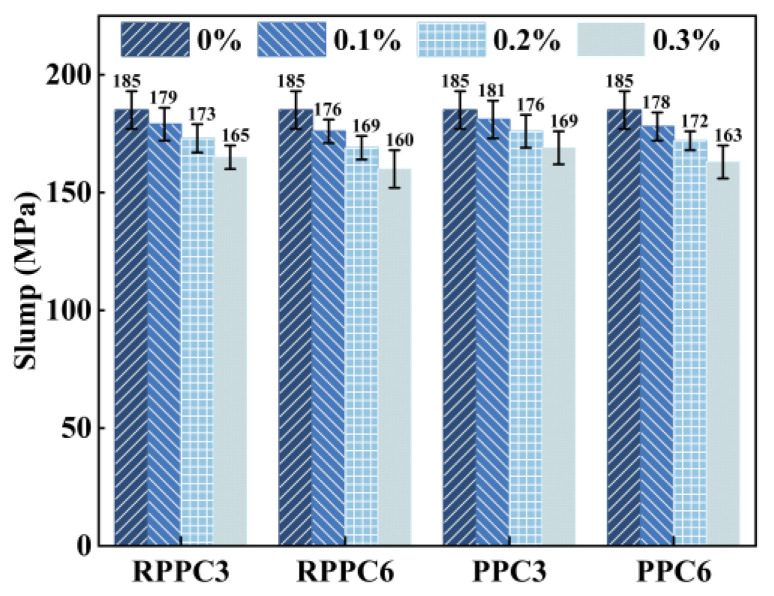
Slump of different fiber-reinforced concretes.

Figure 4 shows the slump of concrete with different fibers. As shown in the figure, compared with the baseline group, the slump of all concrete groups with added fibers decreased, and the slump gradually decreased with the increase in fiber content. This indicates that the addition of fibers increases the frictional resistance inside the fresh concrete and restricts the free flow of cement paste and aggregate to a certain extent, which is consistent with the study of Zhang [27], where the slump decreases more and more significantly with the increase in fiber volume fraction. Under the same fiber type and volume fraction, the slump of concrete with 6 mm fiber is slightly lower than that of concrete with 3 mm fiber. This may be because longer fibers are more likely to overlap or entangle in the matrix, resulting in reduced dispersion uniformity and thus increasing the flow resistance of the mixture. In addition, under the same fiber length and content, the slump of RPP fiber concrete is slightly lower than that of PP fiber concrete, which may be related to the smaller diameter of RPP fibers. Smaller fiber diameter increases the number of fibers per unit volume under the same volume fraction, making it easier to form a denser fiber network in the matrix, thus restricting the fluidity of fresh concrete. Although the addition of fibers reduced the slump of plain concrete from 185 mm to 160–181 mm, no obvious segregation, bleeding, or severe fiber agglomeration was observed during casting. Therefore, the mixtures were considered to have acceptable workability for laboratory casting and vibration compaction under the conditions of this study.

### 3.2. Compressive Performance Analysis

#### 3.2.1. Apparent Peak Strain and Compressive Stress–Strain Response

The apparent strain value corresponding to the peak stress was calculated from the crosshead displacement recorded by the testing machine and normalized by the specimen height. Therefore, it is referred to as the apparent peak strain. This parameter should be interpreted only as a relative indicator of deformation response under the same testing configuration, rather than as a directly measured material strain. The apparent peak strain values of the four fiber-reinforced concretes cured for 7 and 28 days are summarized in Table 5.

For RPPC3 cured for 28 days, increasing the RPP fiber content from 0% to 0.1% increased the apparent peak strain from 0.61% to 0.73%, corresponding to a relative increase of approximately 19%. This result suggests that an appropriate amount of short RPP fibers may improve the apparent deformation response and failure integrity of concrete under compression. This improvement is likely related to the relatively uniform fiber dispersion at low dosage, which helps restrain microcrack development and delays localized damage. However, when the RPP fiber content was further increased to 0.3%, the apparent peak strain decreased to 0.60%. This indicates that excessive fiber addition may reduce the beneficial effect of fiber reinforcement. At higher dosages, fibers are more likely to interlock, entangle, or form local agglomerates, which may introduce additional pores and weak regions in the matrix. These defects can promote stress concentration and accelerate crack initiation and propagation, thereby reducing the apparent deformation response of concrete under the present testing configuration [28,29,30,31,32].

Fiber type and length also influenced the apparent peak strain of concrete. Among the tested mixtures, RPPC3 showed the most evident increase, with the apparent peak strain increasing by approximately 19% compared with PC at 28 days. In contrast, PPC6 showed only a slight increase of approximately 1%, whereas RPPC6 and PPC3 decreased by approximately 3% and 8%, respectively. These differences should not be attributed to a single factor. Instead, they are likely associated with the combined effects of fiber diameter, aspect ratio, surface morphology, number of fibers per unit volume, and dispersion uniformity. Compared with PP fibers, the RPP fibers used in this study had a smaller diameter, which resulted in a larger number of fibers at the same volume fraction. This may help form a denser fiber network and increase the probability of fibers crossing potential crack paths. In addition, the surface morphology of RPP fibers may improve mechanical interlocking with the cementitious matrix to some extent, although this interpretation requires further confirmation by direct interfacial tests.

The aspect ratio of fibers is another important factor affecting fiber dispersion and crack-bridging efficiency [33,34]. A relatively high aspect ratio may enhance crack-bridging capacity, but it can also increase the risk of fiber entanglement and nonuniform dispersion. Conversely, a relatively low aspect ratio may improve dispersion but may provide insufficient embedment length for effective crack bridging and load transfer. Therefore, the reinforcing effect of fibers depends on the balance between dispersion uniformity and mechanical bridging efficiency. In the present study, RPPC3 appeared to achieve a more favorable balance between these two effects, which may explain its superior apparent deformation response under compression. Nevertheless, because direct fiber pull-out tests and interfacial bond measurements were not conducted, the proposed mechanism should be regarded as a reasonable interpretation based on the mechanical results, failure morphology, and SEM observations rather than a direct measurement of interfacial bond strength.

**Figure 5:**
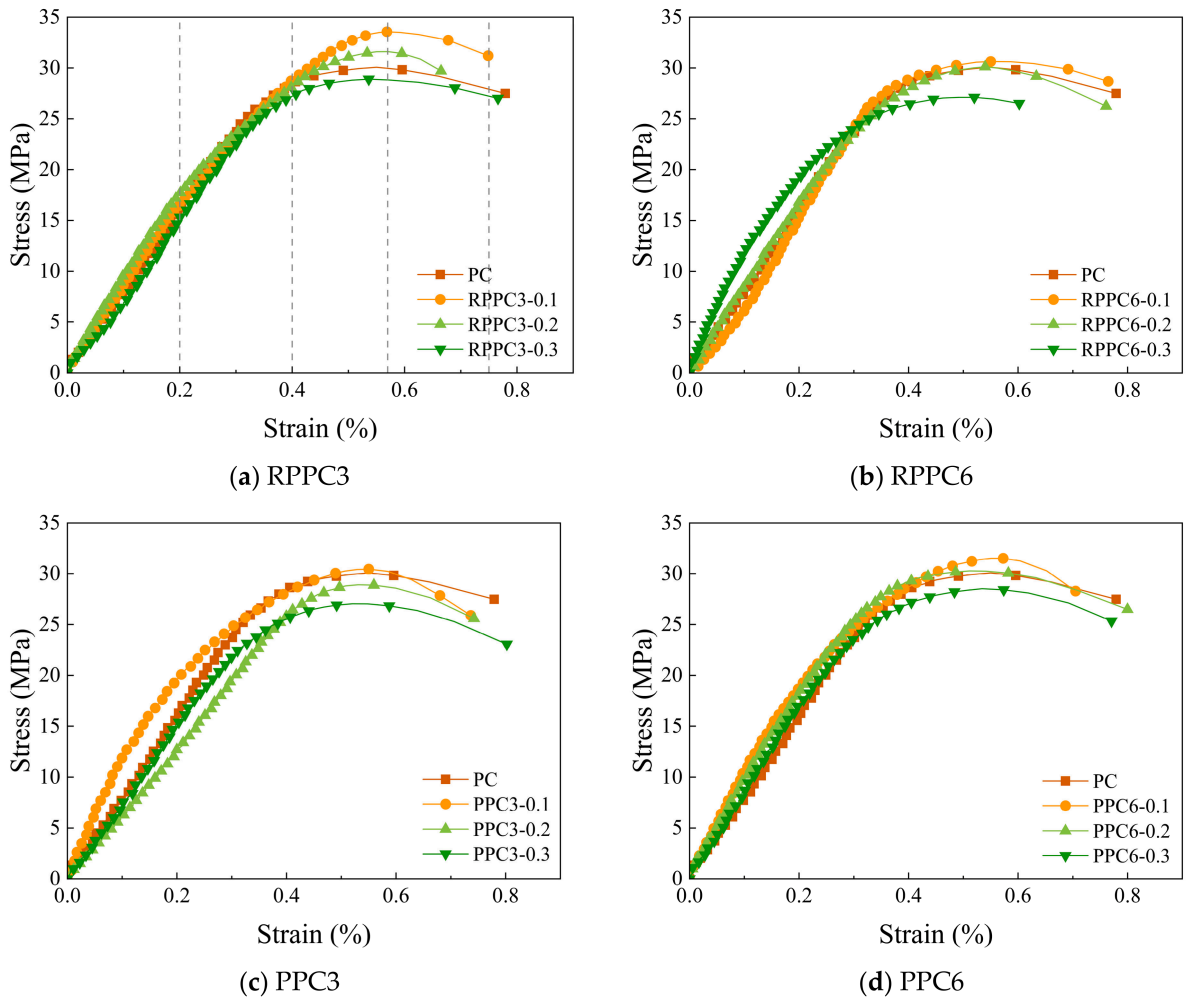
Apparent compressive stress–strain curves of concrete with different fiber contents at 7 d.

**Figure 6:**
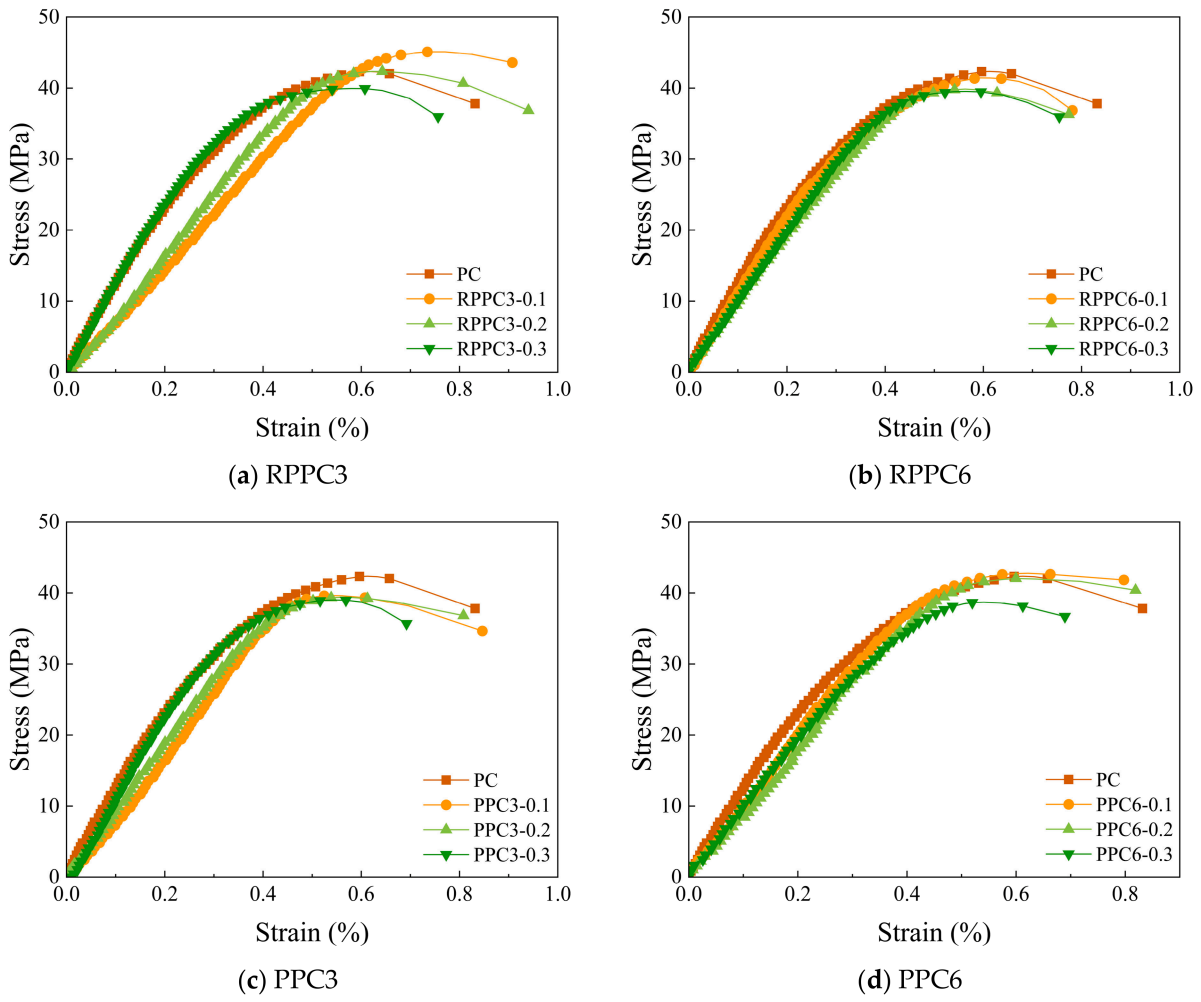
Apparent compressive stress–strain curves of concrete with different fiber contents at 28 d.

The apparent compressive stress–strain curves of the four fiber-reinforced concretes are presented in Figure 5 and Figure 6 for curing ages of 7 and 28 days, respectively. Before formal loading, a small preload was applied to ensure close contact between the loading platen and the specimen surface and to reduce initial seating gaps. It should be noted that the strain values in these curves were calculated from the crosshead displacement recorded by the testing machine; therefore, they may include machine/frame compliance, platen deformation, seating effects, and local contact deformation. Accordingly, the curves are referred to as apparent stress–strain curves and are used only for relative comparison of the deformation response and damage evolution of different mixtures tested under the same conditions.

The differences observed in the initial stage of Figure 5c may be related to the local stiffness changes caused by the random distribution of aggregates, the subtle differences in the surface smoothness of the specimen, and the base effect in the early loading stage. Therefore, the initial slope of the apparent stress–strain curve was not used to determine the elastic modulus. Taking the RPPC3-0.1 curve in Figure 5a as an example, the apparent compressive response can be qualitatively divided into four stages [35]. The first stage is the initial approximately linear response stage, in which the load increases but the visible damage is limited. The second stage corresponds to the stable development stage of microcracks, in which internal microcracks begin to propagate, and fine cracks may gradually appear on the surface of the specimen. The third stage is the crack accelerated propagation stage. As the stress level further increases, the cracks gradually develop and connect with each other in the matrix, the transverse deformation becomes more obvious, and visible short cracks appear on the surface of the specimen. When approaching the peak load, the cracks gradually propagate and form the main failure surface, while the fibers play the role of crack bridging and restraint. The fourth stage is the post-peak failure stage, in which the bearing capacity decreases, and visible edge cracks or spalling phenomena become more obvious. RPPC3-0.1 was used only as a representative example. The same qualitative four-stage apparent compressive response was generally observed for all mixtures at both 7 and 28 days, including the initial approximately linear response, stable microcrack development, accelerated crack propagation, and post-peak failure stages. However, the prominence of each stage and the corresponding failure morphology differed with curing age, fiber type, fiber length, and fiber content. Compared to ordinary concrete, fiber-reinforced specimens generally exhibited more dispersed cracks and better post-peak integrity, indicating that fiber incorporation helps delay localized failure and improve crack control performance.

#### 3.2.2. Compressive Strength

**Figure 7:**
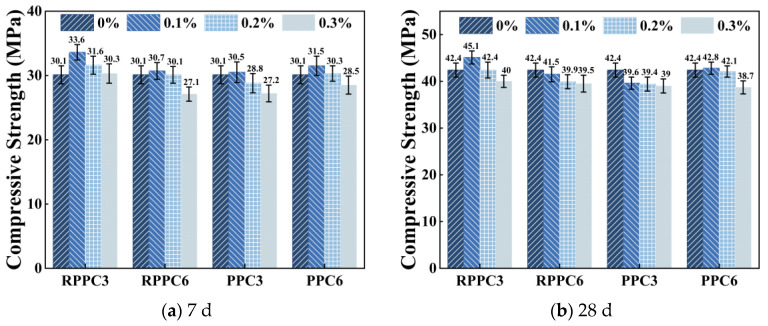
Compressive strengths of concretes at different ages.

The compressive strength values of the four fiber-reinforced concretes after curing for 7 days and 28 days are presented in Figure 7. As shown in Figure 7a, for all specimens, the compressive strength initially increases and then decreases as fiber content rises from 0.1% to 0.3% at 7 d. In contrast, Figure 7b shows a more divergent response at 28 d. RPPC3 and PPC6 still exhibit an initial increase followed by a decrease in compressive strength with increasing fiber content, whereas RPPC6 and PPC3 show a gradual reduction as fiber content increases. This age-dependent difference is likely associated with the combined effects of cement hydration, fiber dispersion, fiber geometry, and fiber–matrix interaction. At 7 d, cement hydration is still incomplete, and both the matrix structure and the fiber–matrix interface are still developing. Therefore, the contribution of fibers to crack restraint and stress transfer is relatively limited. Meanwhile, fiber incorporation may locally disturb the packing structure of cement paste and aggregates, leading to weak interfacial regions or additional microdefects, which may affect early strength development.

As curing progresses to 28 d, the hydration degree increases, the matrix becomes denser, and the interaction between fibers and the cementitious matrix becomes more effective. Under these conditions, the influence of fibers on compressive strength depends not only on fiber content but also on dispersion uniformity, fiber geometry, and the quality of the interfacial transition zone. When the fiber content remains within an appropriate range, fibers can be relatively well dispersed in the matrix and help restrain the initiation and propagation of microcracks through a bridging effect, thereby improving the mechanical performance of concrete. However, excessive fiber content increases the possibility of fiber interlocking, entanglement, and local agglomeration. These agglomerates may weaken the effective contact between fibers and the cement matrix, introduce additional pores and local defects, and cause stress concentration. As a result, the reinforcing effect of fibers may be partially or even completely offset, leading to a reduction in compressive strength [36,37].

Figure 7b further illustrates the differences in peak compressive strength of the four fiber-reinforced concrete types at 28 days. Overall, the effects of different fiber types and lengths on concrete compressive strength vary significantly, with RPPC3 exhibiting the best reinforcement effect, increasing compressive strength by approximately 6% compared to PC. The advantage of RPPC3 is likely related to its smaller fiber diameter, higher aspect ratio, and better dispersion. At the same volume fraction, a smaller fiber diameter means more individual fibers can be distributed per unit volume of concrete, increasing the probability of fibers traversing potential microcrack paths and forming a more uniform three-dimensional fiber network [38]. This network helps limit the initiation and propagation of microcracks, promotes local stress redistribution, and thus improves the compressive properties of concrete. In contrast, the compressive strength of PPC3 is approximately 7% lower than that of PC. Although 3 mm PP has a shorter length, theoretically beneficial for dispersion, its larger diameter and lower aspect ratio result in a relatively smaller number of fibers per unit volume at the same volume fraction, leading to a less dense crack-constraining network compared to RPP. Meanwhile, PP, being a hydrophobic polymer fiber, has limited interfacial compatibility with the cement matrix, potentially weakening its stress transfer and crack restraint effects during compressive failure [39]. Therefore, PPC3 failed to exhibit a significant compressive strengthening effect.

For the 6 mm fiber group, RPPC6 showed a slight decrease in compressive strength compared to PC, while PPC6 showed only a slight increase. Compared to 3 mm fibers, 6 mm fibers have a longer embedment length, theoretically beneficial for crack bridging; however, longer fibers are more prone to overlapping, entanglement, or local aggregation during mixing, reducing their dispersion uniformity in the matrix and potentially introducing local porosity and weak areas. Especially with increased fiber length, the effect of fibers on the flowability of fresh concrete becomes more pronounced, leading to decreased internal uniformity and density of the specimen, thus offsetting its potential strengthening effect. Therefore, the compressive strength improvement of the 6 mm fiber group was not significant.

In summary, the influence of fibers on the compressive strength of concrete is not solely determined by fiber type or surface morphology, but is jointly controlled by fiber geometry, dispersion uniformity [17], fiber quantity per unit volume, workability of the mixture, and the risk of fiber agglomeration. Within the experimental scope of this paper, RPPC3 can form a relatively uniform and dense fiber distribution network at lower dosages, while avoiding significant entanglement and agglomeration problems caused by long fibers. Therefore, RPPC3 exhibits the best compressive strength enhancement effect.

To evaluate the repeatability of the compressive strength results, the standard deviation and coefficient of variation were calculated based on three parallel specimens for each mixture, as shown in Table 6 and Table 7. The CV values of compressive strength ranged from 1.07% to 8.77% at 7 d and from 2.65% to 8.92% at 28 d. All CV values were lower than 10%, indicating acceptable repeatability of the compressive strength tests. The relatively larger variability observed in several fiber-reinforced mixtures, such as PPC6-0.1 at 7 d and RPPC6-0.1 and RPPC6-0.3 at 28 d, may be associated with the random distribution of fibers, local fiber entanglement, and heterogeneity of the fiber–matrix interface. Overall, the experimental variability did not affect the main trend that fiber type, length, and content jointly influenced the compressive strength of concrete.

#### 3.2.3. Failure Morphology

**Figure 8:**
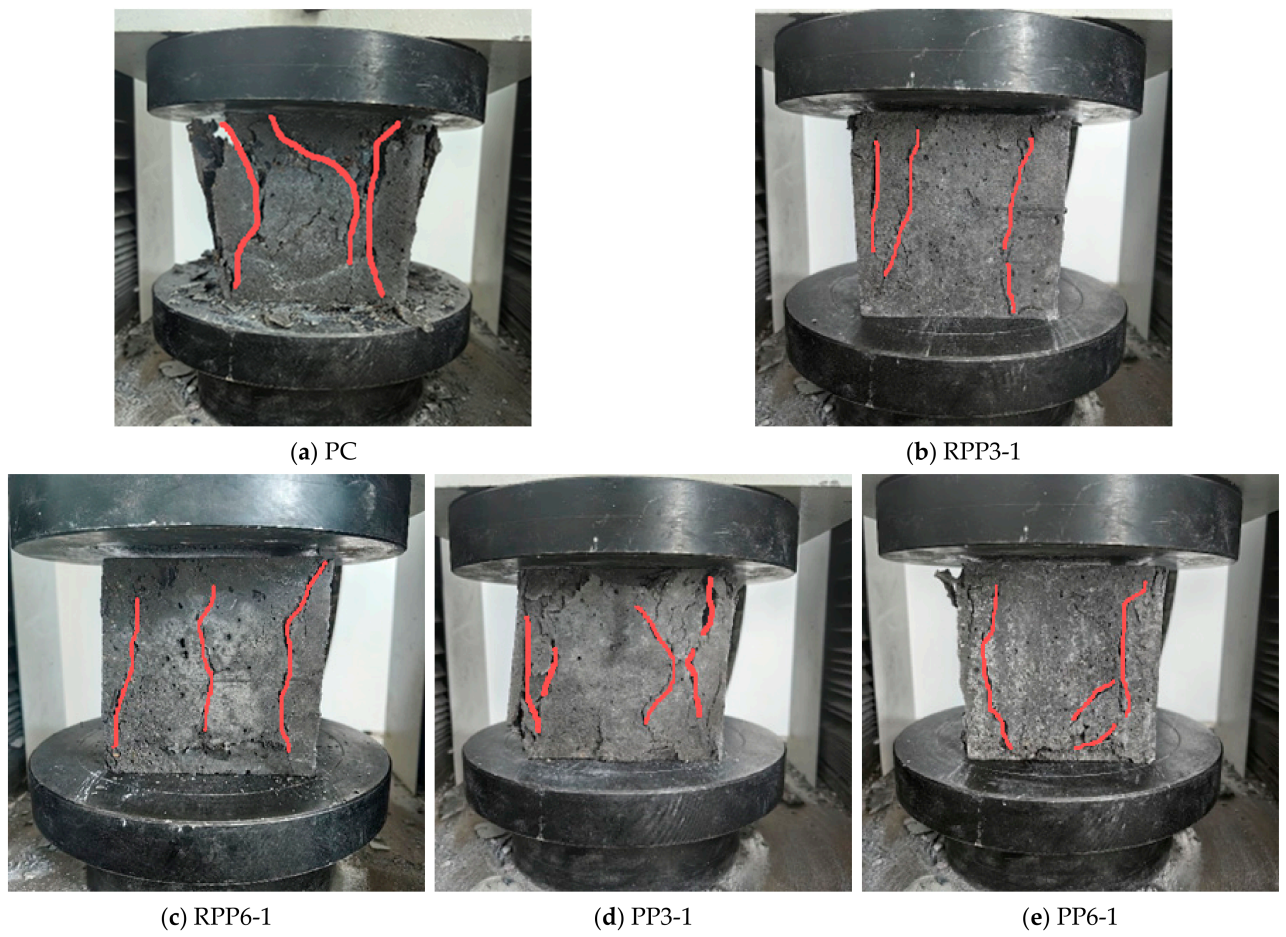
Crack patterns of concrete specimens under failure‌.

To investigate the effect of failure morphology, this study captured failure images of specimens during compressive tests and analyzed their crack evolution. Representative crack patterns at failure are presented in Figure 8. Due to the large number of tested specimens, only those with optimal fiber content are displayed. PC specimens showed classic brittle failure: near peak load, cracks rapidly localized into a few dominant crack zones, with propagation along straight, co-linear, through-going paths [40]. Severe surface spalling accompanied this process, culminating in complete longitudinal splitting. This behavior stems from the inherent tendency of PC to undergo rapid, synchronized growth of micro-cracks under compressive loading. Once a primary crack path forms, intense local stress concentration accelerates crack penetration along the weakest plane, triggering spallation and resulting in abrupt energy release, indicating very low ductility. In contrast, fiber incorporation significantly alters the failure morphology: surface spalling is substantially reduced, and macroscopic crack widths are significantly narrower [40,41]. Simultaneously, the total number of cracks increases, and their spatial distribution becomes markedly more irregular and dispersed. This transformation is attributed to a “multi-scale confinement” effect exerted by fibers throughout the failure process. First, fibers form a randomly oriented, three-dimensional bridging network within the matrix which can provide cross-crack constraints during the initiation stage of micro-cracks, inhibit early crack growth and delay the rapid convergence of micro-cracks into macro-cracks, thereby enhancing the matrix toughness [41]. Second, as cracks propagate further, fibers continuously consume energy through interfacial bonding–friction–pullout mechanism, which requires higher external work for crack propagation, weakens the dominant position of a single primary crack and promotes the cracks to develop in a dispersed manner of “multi-point initiation and multi-path propagation” pattern, manifested as finer, denser, and more randomly oriented cracks. These observations align with prior experimental and theoretical findings reported in the literature [42]. Owing to effective limitation of cracks, the surface concrete is less likely to show large-scale spalling during the peak and post-peak stages, presenting a more stable failure process and higher residual load-bearing capacity macroscopically.

Crack patterns transition from “few, wide, and vertically penetrating” to “numerous, narrow, and irregularly distributed”, indicating a fundamental transition in internal damage localization from concentrated to dispersed. This dispersion mitigates local stress peaks, delays the onset of global instability, and promotes a ductile-like failure response. Moreover, fiber reinforcement constrains crack propagation by forcing path deflection and bifurcation, thereby increasing crack tortuosity and total fracture surface area, which significantly enhances energy dissipation during failure accumulation. RPP3 fibers, due to their superior dispersion homogeneity and more uniform three-dimensional bridging network, enable more effective inhibition of failure dispersion and restriction of crack extension. Consequently, specimens incorporating RPP3 fibers consistently display finer, more densely distributed cracks with markedly reduced through-going penetration, which is directly correlated with their elevated compressive strength.

### 3.3. Analysis of Splitting Tensile Strength

#### 3.3.1. Apparent Splitting Tensile Stress–Strain Response

**Figure 9:**
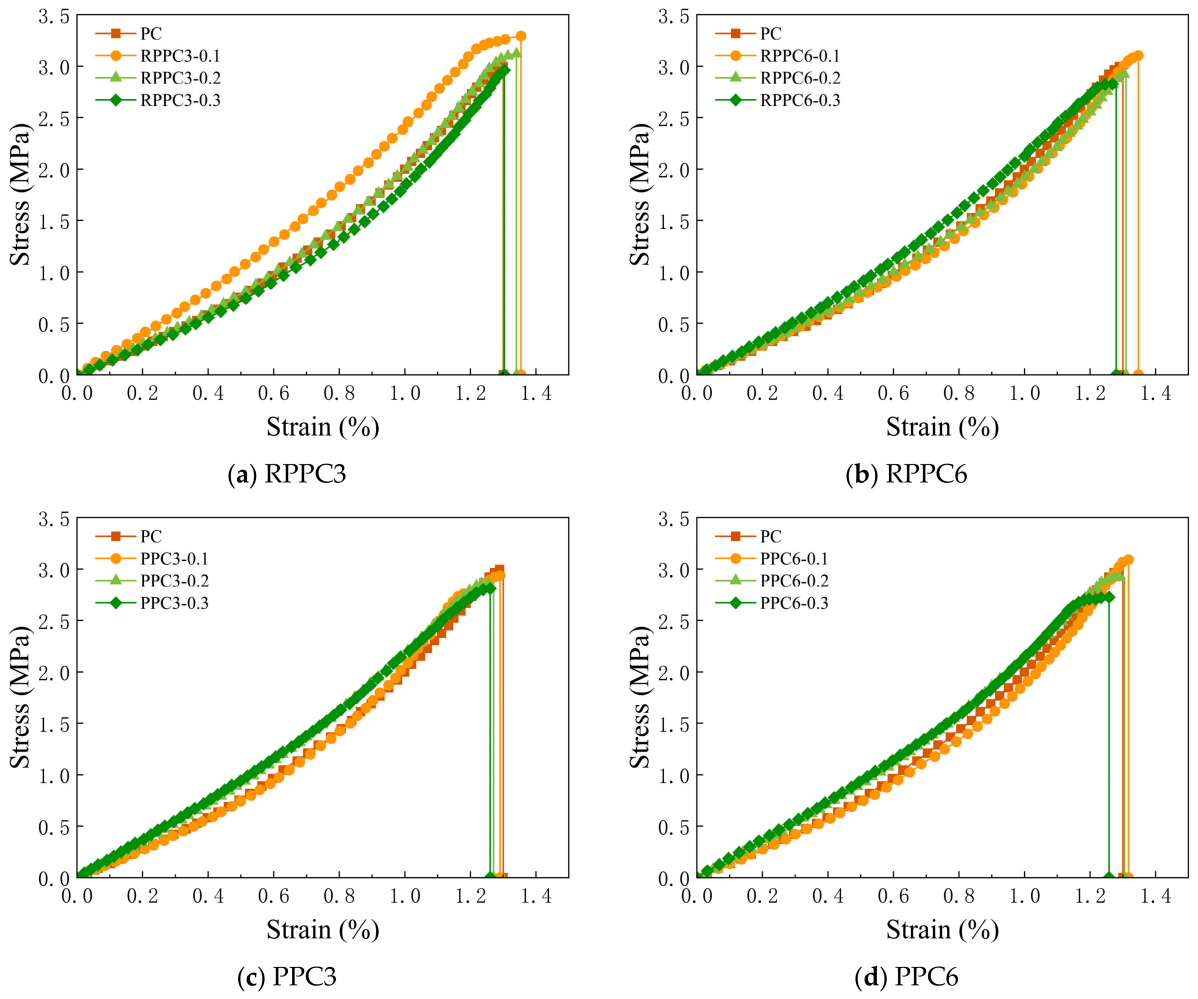
Apparent splitting tensile stress–strain curves of concrete with different fiber contents at 7 d.

**Figure 10:**
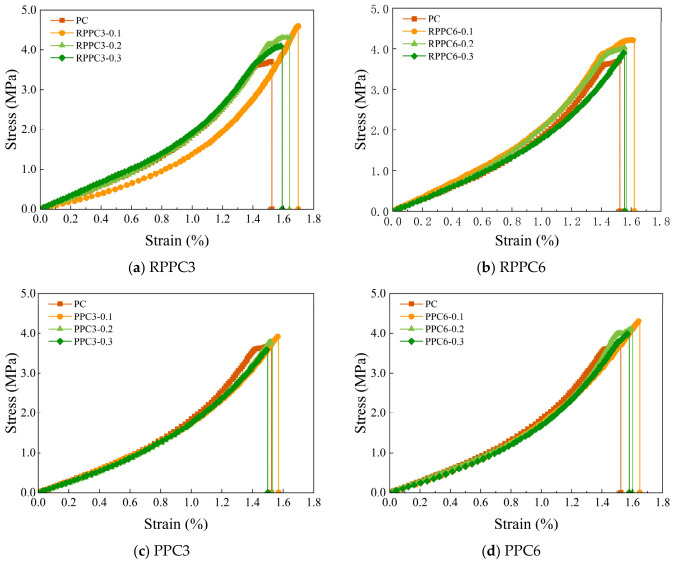
Apparent splitting tensile stress–strain curves of concrete with different fiber contents at 28 d.

The apparent splitting tensile stress–strain curves of the four fiber-reinforced concretes with different fiber contents are shown in Figure 9 and Figure 10. Similar to the compressive tests, the strain values were calculated from the crosshead displacement recorded by the testing machine and normalized by the specimen height. Therefore, these curves are referred to as apparent stress–strain curves and are used only for relative comparison of the global deformation response during splitting tensile loading, rather than for determining intrinsic tensile strain, tensile modulus, or fracture energy.

Under splitting tensile loading, tensile stress is mainly generated along the vertical loading diameter of the cubic specimen. The apparent splitting tensile response can be qualitatively divided into three stages [43]. The first stage is the initial compaction and approximately linear response stage, during which initial pores, microvoids, and weak interfacial regions are gradually closed [44]. The second stage is the stable microcrack initiation and propagation stage. As tensile stress increases, microcracks develop around weak regions such as interfacial transition zones, capillary pores, and fiber–matrix interfaces [31]. In fiber-reinforced concrete, fibers crossing potential crack paths can transfer part of the tensile stress through interfacial friction, mechanical restraint, and pull-out resistance, thereby delaying crack coalescence. The third stage is the unstable crack coalescence stage. At this stage, distributed microcracks connect to form the main splitting crack, while fibers bridging the crack surfaces may continue to resist crack opening through debonding, slippage, pull-out, or fracture [43]. These qualitative mechanisms are consistent with improved crack-bridging behavior and a more stable failure process than that of plain concrete.

The differences among the four fiber-reinforced concretes are mainly related to fiber geometry and dispersion characteristics. SEM observations showed that both PPF and RPPF had relatively smooth surfaces; therefore, the difference in splitting tensile performance should not be attributed solely to surface roughness. As shown in Table 1, RPPF had a smaller diameter and higher aspect ratio than PPF at the same nominal length. At the same volume fraction, this allows more individual RPPF fibers to be distributed in the matrix, increasing the probability of fibers crossing potential crack paths and improving crack-bridging efficiency. In addition, 3 mm fibers are generally easier to disperse uniformly than 6 mm fibers, whereas excessive fiber content may reduce workability and increase the risk of local agglomeration. Therefore, the superior performance of RPPC3-0.1 is likely associated with the combined effects of smaller fiber diameter, relatively high aspect ratio, sufficient fiber number per unit volume, and good dispersion at low fiber content.

#### 3.3.2. Splitting Tensile Strength

**Figure 11:**
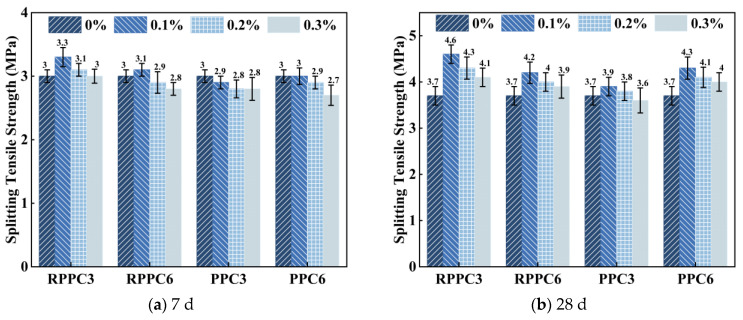
Splitting tensile strength of concretes at different ages.

Figure 11 shows the splitting tensile strength of the four fiber-reinforced concretes after curing for 7 and 28 days. As shown in Figure 11a, for RPP fiber-reinforced concrete, splitting tensile strength initially rises and then declines with increasing fiber content at 7 days. This non-monotonic trend arises because hydration remains incomplete at this early stage, yielding a relatively porous and weak matrix. Under these conditions, the negative impact of fibers on the strength of the matrix is more significant. In Figure 11b, at 28 days, the splitting tensile strength of concrete generally shows a trend of first increasing and then decreasing. This is because at 28 days, cement hydration is substantially complete, leading to marked improvement in fiber–matrix interfacial adhesion. Consequently, fibers effectively redistribute localized stress and delay both crack initiation and propagation. However, once fiber content exceeds the critical threshold of 0.1%, fibers tend to agglomerate within the concrete matrix [32,45], inducing localized porosity and stress concentration, thereby ultimately reducing splitting tensile strength.

Although all four fiber-reinforced concretes exhibit a non-monotonic trend of firstly increasing and then decreasing in splitting tensile strength at 28 days, the effects of fiber types in improving concrete strength differ markedly. Among them, RPPC3 showed the most significant improvement, with a strength increase of 24% compared to PC. This advantage stems from two synergistic effects: (1) RPP fibers may provide more effective mechanical restraint across crack surfaces through interfacial friction and mechanical interlocking [46], especially in the interfacial transition zone, thereby significantly inhibiting fiber pull-out under load and forming a stronger connection. (2) The 3 mm long fibers have good dispersion during mixing, minimizing the risk of entanglement, enabling them to contact hydration products uniformly, and promoting uniform spatial distribution, thus making stress transfer in the matrix more effective and uniform.

RPPC6 achieves a 13% strength gain, primarily attributed to its longer fiber length, which enhances bridging capacity across wider crack openings. However, due to the long length of the 6 mm fiber, more tension occurs during the stress process. Although it can effectively block the expansion of cracks and absorb energy, long fibers are prone to entanglement during the mixing process, resulting in uneven distribution in the matrix, thereby forming stress concentration.

The improvement in splitting tensile strength of PPC3 is relatively limited, only 5% higher than that of PC. Although 3 mm PP also has good dispersion potential, its fiber diameter is larger than that of RPP, resulting in fewer fibers per unit volume for the same volume fraction and a lower density of the fiber bridging network. Furthermore, PP is a hydrophobic polymer fiber, and its interfacial compatibility with the cement matrix is relatively limited, potentially affecting stress transfer and fiber bridging efficiency during crack propagation. Therefore, PPC3’s improvement in splitting tensile strength is relatively small.

PPC6’s splitting tensile strength is approximately 16% higher than that of PC, and its reinforcing effect is higher than both PPC3 and RPPC6. For PP, 6 mm fibers have a longer embedment length and a larger matrix contact range compared to 3 mm fibers, providing more effective cross-crack restraint during crack propagation, thus improving splitting tensile strength. However, compared to RPPC3, PPC6 still has some risk of uneven dispersion and local entanglement; therefore, its reinforcing effect is still lower than that of RPPC3. Therefore, RPPC3 exhibits the best effect in improving tensile strength.

The variability of splitting tensile strength was also evaluated using the standard deviation and coefficient of variation, as summarized in Table 8 and Table 9. The CV values ranged from 1.84% to 8.02% at 7 d and from 1.26% to 9.49% at 28 d. All CV values were below 10%, suggesting that the splitting tensile strength results also had acceptable repeatability. Compared with compressive strength, splitting tensile strength showed slightly higher variability in some 28 d mixtures, such as PPC3-0.1, PPC3-0.2, RPPC6-0.1, and RPPC6-0.2. This is reasonable because tensile failure is more sensitive to local defects, crack initiation sites, and the random distribution of fibers across the splitting plane. Nevertheless, the overall strength trend remained consistent among parallel specimens, supporting the reliability of the observed effects of fiber type, fiber length, and fiber content on splitting tensile performance.

### 3.4. Micromorphological Analysis

**Figure 12:**
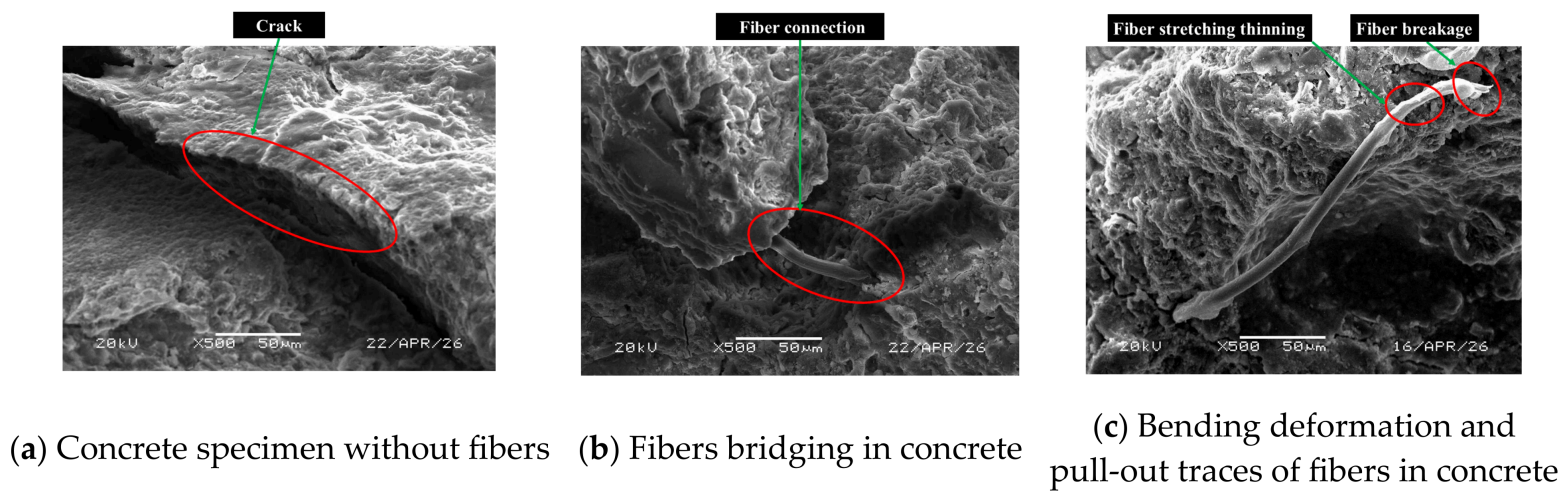
SEM microstructure of the concrete baseline group and the recycled fiber group.

To further investigate the role of short-cut recycled polypropylene fibers in the concrete matrix, the microstructure of the damaged specimens after 28 days of curing was characterized by scanning electron microscopy (SEM), as shown in Figure 12. Figure 12a shows the plain concrete specimen without fibers. Relatively wide and continuous microcracks can be observed in the matrix, which is consistent with the macroscopic failure morphology discussed above. This indicates that, in the absence of fiber restraint, cracks are more likely to localize and propagate into dominant main cracks under mechanical loading.

Figure 12b shows fibers bridging microcracks in the cementitious matrix. This observation suggests that fibers can provide physical restraint across crack surfaces and help limit further crack opening. The interaction between the fibers and the surrounding cement paste may involve interfacial friction, mechanical interlocking, and partial debonding during crack propagation [47,48]. However, because direct pull-out tests or interfacial shear tests were not conducted in this study, the interfacial bonding mechanism is discussed here as a qualitative interpretation based on SEM observations. Figure 12c shows bending deformation and pull-out traces of fibers on the failure surface. These features indicate that, during crack development, fibers may participate in stress transfer and energy dissipation through deformation, debonding, sliding, pull-out, or fracture. Such mechanisms can delay crack opening and reduce sudden crack propagation, which helps explain the improved crack-control behavior and the enhancement in compressive and splitting tensile strengths observed in the fiber-reinforced specimens [49,50].

## 4. Conclusions and Outlook

### 4.1. Conclusions

This study systematically investigated the effects of fiber type, fiber length, and fiber volume fraction on the workability, compressive strength, splitting tensile strength, apparent stress–strain response, failure morphology, and microstructural characteristics of PP- and RPP-fiber-reinforced concrete. The main conclusions are as follows:

(1) The incorporation of both PP and RPP fibers improved the failure morphology and crack-control behavior of concrete. Compared with plain concrete, fiber-reinforced specimens exhibited narrower and more dispersed cracks, reduced surface spalling, and better failure integrity. These improvements can be attributed to the crack-bridging effect of fibers, which helps restrain crack opening and delay localized crack propagation.
(2) Fiber type, length, and volume fraction had different effects on the mechanical properties of concrete. Among all mixtures, RPPC3-0.1 exhibited the best overall performance. At 28 days, its compressive strength and splitting tensile strength were approximately 6% and 24% higher than those of plain concrete, respectively. The improvement in splitting tensile strength was more pronounced than that in compressive strength, indicating that short-cut RPP fibers are more effective in enhancing tensile-related crack resistance.
(3) Within the experimental range of this study, 0.1% was the most favorable fiber volume fraction. When the fiber content increased beyond this level, the mechanical properties tended to decrease to varying degrees. This reduction may be associated with reduced workability, nonuniform fiber dispersion, local fiber entanglement or agglomeration, and additional weak regions in the matrix.
(4) SEM observations further supported the mechanical results. Fiber bridging, bending, and pull-out traces were observed on the failure surfaces, suggesting that fibers can participate in stress transfer and energy dissipation during crack development.

### 4.2. Outlook

The innovation of this paper is mainly reflected in (1) the difference in materials, RPP and PP micron-sized fibers, which are less common in existing research. (2) Through experimental research, it was found that the mechanical properties of concrete are enhanced when the aspect ratio is within a reasonable range. (3) By comparing different fiber dosages, the appropriate dosage range for micron-sized fibers was found.

However, there are also limitations. Flexural tensile strength, residual flexural toughness, crack opening displacement, plastic shrinkage and drying shrinkage, durability in corrosive environments, and high-temperature spalling performance were not included in this experimental protocol and should be investigated in future work. In particular, flexural testing can more directly assess post-cracking performance and residual load-bearing capacity, while shrinkage and high-temperature testing help clarify whether RPP fibers have similar advantages to traditional PP fibers in crack control and fire-resistance applications. Future research should also examine higher fiber volume fractions and wider aspect ratio ranges, because while increasing fiber content can improve fiber bridging density and shrinkage control, it may also reduce processing performance, increase porosity, promote fiber agglomeration, and decrease compressive or tensile strength beyond the optimal range.

## Figures and Tables

**Table 1:** Particle size distribution of fine aggregate.

Particle Size (mm)	2.36–4.75	1.18–2.36	0.60–1.18	0.30–0.60	0.15–0.30	<0.15
Percentage (%)	6	17	27	33	12	5

**Table 2:** Particle size distribution of coarse aggregate.

Particle Size (mm)	16–20	10–16	5–10	<5
Percentage (%)	18	42	36	4

**Table 3:** Key physical properties of fibers.

Fiber Type	Diameter (μm)	Length (mm)	Aspect Ratio	Breaking Strength (MPa)	Elastic Modulus (MPa)
RPP	15	3	200	327	3940
		6	400		
PP	39	3	83	390	5300
		6	166		

**Table 4:** Mix proportion design and numbering of concrete.

Specimen No.	Cement (kg/m^3^)	Coarse Aggregate (kg/m^3^)	Fine Aggregate (kg/m^3^)	Water (kg/m^3^)	Fiber (kg/m^3^)
PC	380.0	1178.0	676.4	190.0	0
PPC3-0.1	380.0	1178.0	676.4	190.0	0.9
PPC3-0.2	380.0	1178.0	676.4	190.0	1.8
PPC3-0.3	380.0	1178.0	676.4	190.0	2.7
PPC6-0.1	380.0	1178.0	676.4	190.0	0.9
PPC6-0.2	380.0	1178.0	676.4	190.0	1.8
PPC6-0.3	380.0	1178.0	676.4	190.0	2.7
RPPC3-0.1	380.0	1178.0	676.4	190.0	0.9
RPPC3-0.2	380.0	1178.0	676.4	190.0	1.8
RPPC3-0.3	380.0	1178.0	676.4	190.0	2.7
RPPC6-0.1	380.0	1178.0	676.4	190.0	0.9
RPPC6-0.2	380.0	1178.0	676.4	190.0	1.8
RPPC6-0.3	380.0	1178.0	676.4	190.0	2.7

**Table 5:** Apparent peak strain values of different fiber-reinforced concretes in compressive strength tests.

Fiber Content (%)	RPPC3		RPPC6		PPC3		PPC6	
	7 d	28 d	7 d	28 d	7 d	28 d	7 d	28 d
0	0.54	0.61	0.54	0.61	0.54	0.61	0.54	0.61
0.1	0.56	0.73	0.55	0.59	0.55	0.56	0.55	0.62
0.2	0.55	0.62	0.54	0.56	0.53	0.56	0.54	0.59
0.3	0.53	0.60	0.52	0.56	0.52	0.55	0.53	0.54

**Table 6:** Standard deviations of compressive strength.

Fiber Content (%)	RPPC3		RPPC6		PPC3		PPC6	
	7 d	28 d	7 d	28 d	7 d	28 d	7 d	28 d
0	1.32	2.53	1.32	2.53	1.32	2.53	1.32	2.53
0.1	0.36	3.10	2.18	3.70	2.23	2.59	2.76	1.91
0.2	1.45	1.40	1.95	2.79	2.11	1.12	2.26	1.11
0.3	2.27	1.42	1.08	3.51	0.98	1.65	2.13	1.91

**Table 7:** Coefficients of variation in compressive strength.

Fiber Content (%)	RPPC3		RPPC6		PPC3		PPC6	
	7 d	28 d	7 d	28 d	7 d	28 d	7 d	28 d
0	4.39	6.02	4.39	6.02	4.39	6.02	4.39	6.02
0.1	1.07	6.87	7.10	8.92	7.30	6.55	8.77	4.46
0.2	4.60	3.30	6.48	6.99	7.32	2.85	7.48	2.65
0.3	7.47	3.54	3.99	8.88	3.62	4.24	7.47	4.93

**Table 8:** Standard deviations of splitting tensile strength.

Fiber Content (%)	RPPC3		RPPC6		PPC3		PPC6	
	7 d	28 d	7 d	28 d	7 d	28 d	7 d	28 d
0	0.17	0.26	0.17	0.26	0.17	0.26	0.17	0.26
0.1	0.26	0.06	0.06	0.36	0.10	0.36	0.17	0.26
0.2	0.17	0.36	0.17	0.36	0.06	0.36	0.12	0.06
0.3	0.20	0.36	0.12	0.06	0.15	0.25	0.17	0.26

**Table 9:** Coefficients of variation in splitting tensile strength.

Fiber Content (%)	RPPC3		RPPC6		PPC3		PPC6	
	7 d	28 d	7 d	28 d	7 d	28 d	7 d	28 d
0	5.77	7.15	5.77	7.15	5.77	7.15	5.77	7.15
0.1	8.02	1.26	1.84	8.58	3.45	9.25	5.77	6.15
0.2	5.59	8.39	5.97	9.01	2.09	9.49	3.94	1.40
0.3	6.67	8.79	4.08	1.49	5.52	7.06	6.42	6.61

## Data Availability

The original contributions presented in this study are included in the article. Further inquiries can be directed to the corresponding author.
